# Needs of family caregivers in home care for older adults**^1^**

**DOI:** 10.1590/1518-8345.1511.2870

**Published:** 2017-04-06

**Authors:** Carla Cristiane Becker Kottwitz Bierhals, Naiana Oliveira dos Santos, Fernanda Laís Fengler, Kamila Dellamora Raubustt, Dorothy Anne Forbes, Lisiane Manganelli Girardi Paskulin

**Affiliations:** 2 Doctoral student, Universidade Federal do Rio Grande do Sul, Porto Alegre, RS, Brazil. Scholarship holder at Coordenação de Aperfeiçoamento de Pessoal de Nível Superior (CAPES), Brazil.; 3 Master's student, Universidade Federal do Rio Grande do Sul, Porto Alegre, RS, Brazil.; 4 Undergraduate student in Nursing, Universidade Federal do Rio Grande do Sul, Porto Alegre, RS, Brazil. Scholarship holder at Fundação de Amparo à Pesquisa do Rio Grande do Sul (FAPERGS), Brazil.; 5 PhD, Professor, Faculty of Nursing, University of Alberta, Edmonton, AB, Canada.; 6 PhD, Associate Professor, Escola de Enfermagem, Universidade Federal do Rio Grande do Sul, Porto Alegre, RS, Brazil.

**Keywords:** Nursing, Caregivers, Aged, Primary Health Care, Health Education

## Abstract

**Objective:**

to reveal the felt and normative needs of primary family caregivers when providing instrumental support to older adults enrolled in a Home Care Program in a Primary Health Service in the South of Brazil.

**Methods:**

using Bradshaw's taxonomy of needs to explore the caregiver's felt needs (stated needs) and normative needs (defined by professionals), a mixed exploratory study was conducted in three steps: Descriptive quantitative phase with 39 older adults and their caregiver, using a data sheet based on patient records; Qualitative exploratory phase that included 21 caregiver interviews, analyzed by content analysis; Systematic observation, using an observation guide with 16 caregivers, analyzed by descriptive statistics.

**Results:**

the felt needs were related to information about instrumental support activities and subjective aspects of care. Caregivers presented more normative needs related to medications care.

**Conclusion:**

understanding caregivers' needs allows nurses to plan interventions based on their particularities.

## Introduction

The continuous growth in the number of older adults in the past few years worldwide has resulted in an increase in chronic disease rates. In the Brazilian population, chronic diseases occupy a prevalent role in the list of mortality causes^1^. Thus, a greater number of older adults are dependent on accessing health care professionals and settings. Family members have been shown to play an important role in this context^2^. 

Investigations conducted in developed countries observed that caregivers need to receive information on: the disease process, supporting health care resources and access to health services^3^ ^-^ ^4^. Studies conducted in developing countries like Brazil, however, found that the needs are more related to the performance of instrumental support activities, fear of not providing proper care, family dynamics, lack of information on the health condition, total dedication to care, financial expenses, among others^2^ ^,^ ^5^.

It is worth noting that studies conducted both in developed and developing countries have investigated and questioned the needs of the caregivers, but without exploring whether there is coherence between what they manifested (or said) and what nurses and other professionals observed as a need for the practice of care. Additionally, previous studies have not investigated in detail how caregivers deal with instrumental support for older adults. Instrumental support is defined as the aid to perform activities in general, such as personal care (e.g., bathing, dressing, medications care, excretion, transfer), help with household chores and financial support^6^.

Patients from developed countries rely on a network of well-established formal services, such as day care, home care and long-term care facilities. In developing countries like Brazil, however, given the scarcity or lack of these services, relatives have the main responsibility for providing care to dependent older adults. Consequently, family caregivers could benefit from receiving support, guidance and assistance in caring for a family member^2^.

In health care, the term "need" has several meanings that constantly change^7^. The concept of *needs* used in this study was according to Bradshaw's taxonomy, which proposes four categories to address the different perspectives of health needs, namely: comparative need, expressed need, normative need, and felt need^7^. A *comparative need* results from differences between two groups when comparing the rendering of a given service. An *expressed need* is a perceived need demanded by people seeking to use a service. A *normative need* is defined according to a norm or institutional criterion acknowledged by professionals. Finally, a *felt need* represents individuals' desires and wishes and is limited by the perception of each subject^7^. For this study, two categories from Bradshaw's taxonomy were used: the felt and normative. 

Therefore, the objective of this study was to reveal the felt and normative needs of primary family caregivers when providing instrumental support to older adults enrolled in a Home Care Program (HCP) in a Primary Health Service in the South of Brazil. 

## Methods

### Study design

A mixed exploratory study was conducted in three steps: (1) descriptive quantitative, (2) qualitative exploratory and (3) systematic observation, between June and November 2014 in a Primary Health Care Service (PHCS) in the South of Brazil. The PHCS offers a Home Care Program (HCP) with nurses, family physicians and assistant nurses. 

### Sample

The study population comprised 55 older adults aged 60 years and over participating in the HCP and their primary family caregivers who were responsible for the care of the older adult. The sample varied according to each phase of the study.

In the *descriptive quantitative phase*, of the 55 participants, 39 older adults were intentionally selected with their caregivers, who complied with the inclusion criterion of having a primary family caregiver who performed some instrumental support activity related to personal care for the older adult, according to data obtained from patient records. 

Of the 39 participants identified in the first phase, all were approached by phone and invited to participate in the second phase. However, 18 were excluded according to the following criteria: three caregivers because their relatives died, two because older adults were hospitalized, two family caregivers could not be contacted, seven refused to participate in the study, and four had participated in the pilot test for the preliminary evaluation of the observation guide designed by expert consensus for this study, resulting in 21 who participated in the *exploratory qualitative phase*. This number of participants is adequate for qualitative research^8^.

Of these 21 family caregivers, four refused to participate in the *systematic observation phase* and one family member died during this collection period, resulting in a sample of 16 family caregivers in the last phase. The number of participants is limited for a quantitative study and does not permit generalizations. However, all eligible participants in the study population have participated. 

### Data collection

The first author collected the data for all phases from June 2014 to November 2014. For the *descriptive quantitative phase*, we used the HCP list of patients. The older adult variables were: gender, age, years of schooling, income (minimum wage), morbidities and functional capacity to perform activities of daily living, assessed by the Katz Index and the Lawton Scale^9^. These variables were selected from the records of the HCP. The caregiver variables were: gender, age, relationship with the older adult, and instrumental support activities performed by the caregiver. After analyzing the frequency of performance of these activities, the four most frequent ones, that is, which caregivers performed with a frequency ≥ 55%, were selected, namely: bathing, dressing, diaper changing and medications care.

In the *exploratory qualitative phase*, home visits were made to conduct a semi-structured interview designed for this study, with questions addressing the concerns and difficulties of older adult care related to the four instrumental support activities selected in the first phase of the study. The questions were: How did you learn to perform the instrumental support activity? What were your concerns related to the care? Where did you find information related to your concerns? What difficulties did you experience related to the care? Caregivers' responses represented their views and needs with regard to older adult care.

Subsequently, the *systematic observation phase* was conducted, comprising a new home visit to observe family caregivers performing the instrumental support activities that they reported in the previous phase as the most frequently performed (*bathing, dressing, diaper changing* or *medications care*). It should be highlighted that the activity *bathing* was divided into *bed bathing* and *shower bathing*, with the purpose of understanding all levels of older adult dependency. 

The observations followed a guide designed for this study by expert consensus of nurses experienced in older adult care and HCP. This guide explains the essential care tasks that family caregivers should perform to assist older adults with each instrumental support activity, i.e. the essential knowledge caregivers should have and the basic procedures they should perform in each of these activities. The first author observed the family caregivers when they were performing the instrumental activity of care to the older adults. At the same time, the first author checked if they were performing the essential activities, or if they were not performed or incompletely performed. These essential care activities reported as non-performed or incompletely performed were considered as normative needs of family caregivers.

### Analysis

Data from the first phase of the study were analyzed using the Statistical Package for the Social Sciences (SPSS 18.0) through descriptive statistics. Continuous variables were expressed as mean and standard deviation or median and interquartile range. Categorical variables were expressed as absolute or relative frequencies.

Data from the exploratory qualitative phase were investigated by content analysis, using the following steps: pre-analysis, when the interviews were read; coding of participants' transcribed interviews and development of thematic areas; exploration of material; and interpretation of results^10^. Interviews were read and analyzed by the primary and last researcher independently. Two groups of researchers separately carried out the categorization process for posterior discussion of analysis, which ensures the reliability of the interviews.

The data obtained from observations conducted in the third phase were expressed as absolute and relative frequencies. Subsequently, information provided by caregivers on felt needs (concerns and difficulties in caring for older adults) was combined with data on normative needs (essential activities not performed or incompletely performed) to affirm if both results were in agreement.

### Ethical considerations

The study received approval from the institutional Research Ethics Committee/HCPA No. 140287. The health service team authorized the researchers to collect data from older adults' records and their caregivers of HCP. The researchers signed a liability form for the use of those data. The caregivers and older adults signed an informed consent form, indicating their willingness to participate in the qualitative and observation phase of the study.

## Results

The Table 1 shows the characteristics of the older adults and their family caregivers. 

**Table 1:** Sociodemographic and descriptive characteristics of older adults and their family caregivers from the Home Care Program (HCP). Porto Alegre, RS, Brazil, 2016

Variables		Older adults n=39	Caregivers n=39
Age (years)*		82.9 (±8.9)	59.6 (±12.5)
Sex^†^			
	Females	29 (74.4)	34 (87.2)
Years of education*		6,52 (±3.5)	
Income (minimum wage)*		4(1-14)	
Family relationship with relative^†^			
	Spouse		5 (12.8)
	Child		26 (66.6)
	Sibling		4 (10.2)
	Other		4 (10.2)
Functional capacity			
	Katz Index (Basic Activities of Daily Living)^†^	14 (35.9)	
	G (dependent for all basic activities)	11,37 (±3.0)	
	Lawton Scale (Instrumental Activities of Daily Living)*	2 (1-6)	
Morbidities*			
	High blood pressure^†^	24 (61.5)	
	Stroke^†^	20 (51.3)	
	Dementia^†^	11 (28.2)	
	Diabetes Mellitus^†^	9 (23.1)	

*Continuous variables (mean & SD; range).

†Categorical variables (number & %).

Table 2 shows the instrumental support activities the primary family caregivers most frequently performed. 

**Table 2:** Instrumental support activities primary family caregivers most frequently performed for the older adults from the HCP. Porto Alegre, RS, Brazil, 2016

Instrumental Support Activity	Caregivers n = 39*
Medications care	38 (97,4)
Bathing	26 (66,6)
Diaper changing	22 (56,4)
Dressing	22 (56,4)

*Categorical variables (number & %)

### Exploratory qualitative analysis - felt needs of family caregivers in the care for older adults

To cope with the aim of the study, which was to identify the felt needs of the family caregivers, we selected themes according to the open questions of the interview, related to the instrumental support activities the caregivers performed. Each of the research team members then drew up provisional categories and held a meeting to define the final thematic areas by consensus. Thematic analysis identified the following areas: (1) information on the care provided and (2) difficulties in performing care activities. During the classification of the respondents' statements into different categories within thematic areas, it was observed that these categories were interrelated, i.e. they did not occur in isolation from one another.

### Thematic area 1 - Information on health care

This thematic area included answers related to the questions: How did you learn to perform the instrumental support activity? What were your concerns related to the care? Where did you find information related to your concerns? Most caregivers felt comfortable regarding care activities. This was due to the guidance and follow-up provided by health professionals.

Other caregivers reported to have concerns about devices to facilitate care. Further responses addressed the role of the caregiver and emotional aspects of care, such as understanding the inversion of roles between the caregiver and the older adult.

### Thematic area 2 - Difficulties in performing care activities 

This thematic area corresponded to the question: What were your difficulties related to the care? Caregivers reported the need for a second person to help in the care for the older person; availability of devices to facilitate care; lack of knowledge on the activity to be performed; caregiver health issues; complete dedication to care and impaired social life; and accepting the situation of older adult dependence/inversion of roles. 

### Systematic observation - Normative needs of family caregivers

Table 3 shows the normative needs identified in the systematic observation.

**Table 3:** Instrumental support activities, observations and normative needs of the primary family caregivers to the older adults from the HCP, Porto Alegre, RS, Brazil, 2016

Instrumental support activities	Observations (n)	Normative needs* (%)
Medications care	8	50.00
Dressing	3	50.00
Diaper changing	5	37.50
Shower bathing	3	33.33
Bed bathing	2	18.18

*Categorical variables (number & %).

**Figure 1:**
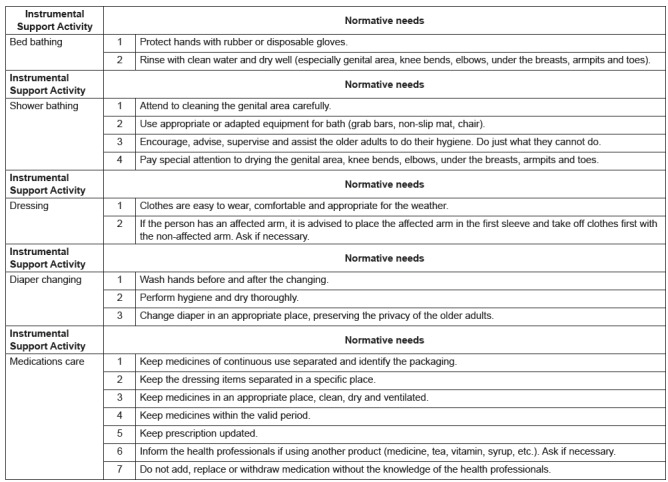
Family caregiver's normative needs for each instrumental support activity to the older adults from the HCP, Porto Alegre, RS, Brazil, 2016

Figure 1 shows the normative needs of family caregivers for each instrumental support activity of older adults' care.

### Integration between felt and normative needs

With regard to the instrumental support activities performed, there was an agreement between the felt need for information and normative needs related to *diaper changing* and *medications care*, since some caregivers were found to have both concerns and normative needs with regard to these activities. Conversely, other caregivers did not report information needs nor had normative needs regarding these activities and *bed bathing*. 

Additionally, there were disagreements between the activities *shower bathing, dressing, diaper changing,* and *medications care,* because some caregivers did not report felt needs for information but did not carry out all the essential care procedures required for the appropriate performance of these activities. 

## Discussion 

### Characteristics of participants 

Mean age, educational level and income for older adults were higher in this study than in other national studies with dependent older adults and their family caregivers^11^ ^-^ ^12^, given that the South of Brazil is one of the most developed regions of the country. As expected, there was a greater proportion of women, in view of the feminization of aging. High dependence to perform instrumental support activities was similar to a study conducted in Northern Brazil with older adults in a HCP^13^. As in another Brazilian study, high blood pressure, stroke and dementia were the most frequent morbidities in older adults dependent on home care^13^.

In line with previous studies in Brazil, family caregivers were mainly middle-aged or older women who were daughters or wives living with the older adult^11^ ^,^ ^14^. The instrumental support activities the caregivers more frequently performed were *medications care* and *bathing*, corroborating findings from a national study conducted with family caregivers and dependent older adults^15^. Conversely, investigations carried out in developed countries show that the instrumental support activities more frequently performed by family caregivers are transportation, accompanying to medical appointments, and care with household chores^16^ ^-^ ^18^. This divergence may be explained by the fact that there is a wider variety of support networks and health care professionals to help caregivers in the performance of more complex instrumental support activities in developed countries when compared to the Brazilian reality.

### Felt and normative needs 

The interviews revealed felt needs for information and/or support for the subjective aspects of care, showing that, in addition to guidance and knowledge on how to perform instrumental support activities of older adult care, the emotional needs of family caregivers should also be considered. One of the issues, the role of the caregiver was also addressed in another Brazilian study^14^. Thus, inversion of role may cause a negative reaction in caregivers, who start to experience contradictory feelings, physical and emotional signs and symptoms, tiredness, and impossibility of performing their own activities because of the dedication to their older adult relative.

Studies conducted in developed countries analyzed information needs, such as access and use of support services and programs, legal and financial support, presence of a support network to help caregivers in caring for older adults safely^2^ ^,^ ^19^. These particularities may be explained by the existence of formal service networks and support programs directed to the care of dependent older adults in developed countries, where care is considered a responsibility not only of families, but also of the society and the state. Unfortunately, this is not a reality in developing countries like Brazil. 

The availability of materials and/or devices to facilitate care was also reported as a challenge in the performance of instrumental support activities. The use of appropriate or adapted equipment for *bathing* was identified as a normative need. Thus, it is necessary not only to guide caregivers, but also to assess the household to identify factors that may contribute to the onset of difficulties in the performance of care. Moreover, caregivers should be instructed on how to have access to this equipment, which the government does not usually offer.

Additionally, caregivers reported difficulties related to their own health issues. Caregiver health problems may be associated with sociodemographic factors, such as female gender, advanced age, degrees of older adult dependence, and lack of skills for the role^20^ ^-^ ^21^. Complete dedication to care and impaired social life were also difficulties identified by family caregivers in another Brazilian study^12^. Social inclusion should be promoted and facilitated by connecting the health services the caregivers visit with the existing social support networks^12^. 

Regarding the activity *medications care*, most caregivers did not report felt needs for information because they received guidance from health care professionals linked to the PHCS. This finding differs from studies in Brazil in which caregivers have a need for information^2^ ^,^ ^22^. Conversely, *medications care* was the activity that had the greatest amount of normative needs in the present study, which reveals that there is a disagreement between caregivers' felt and normative needs. Furthermore, caregivers had a greater amount of normative needs in *medications care* and *dressing*. These results may indicate lack of knowledge on the performance of these activities, probably because health care professionals do not feel the need to give detailed relevant information, given their low complexity. However, this lack of guidance may interfere with the health recovery of older adults. 

Some caregivers reported felt need for information regarding *diaper changing*, a complex care activity not present in the usual routine of some people. Additionally, most caregivers had at least one normative need related to this activity. The complexity of *diaper changing* comes from the fact that caregivers have to deal with the physiological excretions of the older adult. Given these particularities, the appropriate performance of *diaper changing* requires knowledge, preparation, skills and resources^15^.

Caregivers did not report felt need for information regarding the *bed bath*, because of the guidance provided by health care professionals. These professionals were expected to provide guidance on this matter, since a bed bath is a complex care activity that requires knowledge from caregivers and is difficult to perform because of the older adults' limitations.

A study that also used Bradshaw's taxonomy of needs found that caregivers felt that they should receive more support services than is given and have better access to services^4^. The authors investigated these needs in order to evaluate care services available to caregivers and older adults. This approach cannot be systematically adopted in the Brazilian context though. In the present study, felt needs were identified in order to correlate them with normative needs detected in the performance of instrumental support activities. This difference may justify the divergences in felt needs between the two studies.

In Brazil, there is a lack of protocols for the management of home care. Some existing programs propose to evaluate only health care needs of users or the organization of services, but, unlike international protocols, do not systematize care nor suggest interventions. This situation poses a challenge to health services in the planning of actions directed at the needs of dependent older adults and their family caregivers. 

In view of the foregoing, the nursing staff gains importance in the role of "instructing/educating for care", since these staff are directly involved with instrumental support activities during older adult hospitalization and at the PHC level. However, the information provided to caregivers does not exempt or invalidate the participation, responsibility, and care follow-up performed by PHC professionals. Family caregivers should be considered as an integral part of the older adult care system and not as the only provider of care of dependent older adults^23^.

The results will contribute to our understanding of the needs of family caregivers. Additionally, the findings will inform the development of nursing educational programs and interventions directed to their needs for them to be better prepared for this role.

## Conclusions

The caregiver interviews enabled us to identify the felt needs of information for the practice of instrumental support activities. Furthermore, information and/or support about the subjective aspects of care were identified.

Through systematic observation, it was possible to identify the normative needs of each instrumental support activity. In the *bed bath* two normative needs; five for *shower*; three for *dressing*; three for *diaper changing* and seven for *medications care*. The integration of results identified agreement between felt and normative needs for *diaper changing, medications care* and *bed bath*. Disagreements were found in *shower, dressing, diaper changing* and *medications care*. 

The felt and normative needs of family caregivers identified enable nurses to develop care strategies and plan interventions focused on these needs. Moreover, new public policies should be developed and directed to family caregivers, as well as educational programs that support the care and help reduce harm in care for a dependent older adult.
